# Characterizing the Structural Pattern of Heavy Smokers Using Multivoxel Pattern Analysis

**DOI:** 10.3389/fpsyt.2020.607003

**Published:** 2021-02-04

**Authors:** Yufeng Ye, Jian Zhang, Bingsheng Huang, Xun Cai, Panying Wang, Ping Zeng, Songxiong Wu, Jinting Ma, Han Huang, Heng Liu, Guo Dan, Guangyao Wu

**Affiliations:** ^1^Department of Radiology, Panyu Central Hospital, Guangzhou, China; ^2^Medical Imaging Institute of Panyu, Guangzhou, China; ^3^Health Science Center, Shenzhen University, Shenzhen, China; ^4^Shenzhen University Clinical Research Center for Neurological Diseases, Shenzhen, China; ^5^Medical AI Lab, School of Biomedical Engineering, Health Science Center, Shenzhen University, Shenzhen, China; ^6^Department of Radiology, Shenzhen University General Hospital, Shenzhen, China; ^7^Shenzhen University International Cancer Center, Shenzhen, China; ^8^Zhongnan Hospital of Wuhan University, Wuhan, China; ^9^Medical Imaging Center of Guizhou Province, Department of Radiology, The Affiliated Hospital of Zunyi Medical University, Zunyi, China; ^10^School of Biomedical Engineering, Health Science Center, Shenzhen University, Shenzhen, China

**Keywords:** smoking addiction, multivoxel pattern analysis, voxel-based morphometry, machine learning, structural magnetic resonance imaging

## Abstract

**Background:** Smoking addiction is a major public health issue which causes a series of chronic diseases and mortalities worldwide. We aimed to explore the most discriminative gray matter regions between heavy smokers and healthy controls with a data-driven multivoxel pattern analysis technique, and to explore the methodological differences between multivoxel pattern analysis and voxel-based morphometry.

**Methods:** Traditional voxel-based morphometry has continuously contributed to finding smoking addiction-related regions on structural magnetic resonance imaging. However, voxel-based morphometry has its inherent limitations. In this study, a multivoxel pattern analysis using a searchlight algorithm and support vector machine was applied on structural magnetic resonance imaging to identify the spatial pattern of gray matter volume in heavy smokers.

**Results:** Our proposed method yielded a voxel-wise accuracy of at least 81% for classifying heavy smokers from healthy controls. The identified regions were primarily located at the temporal cortex and prefrontal cortex, occipital cortex, thalamus (bilateral), insula (left), anterior and median cingulate gyri, and precuneus (left).

**Conclusions:** Our results suggested that several regions, which were seldomly reported in voxel-based morphometry analysis, might be latently correlated with smoking addiction. Such findings might provide insights for understanding the mechanism of chronic smoking and the creation of effective cessation treatment. Multivoxel pattern analysis can be efficient in locating brain discriminative regions which were neglected by voxel-based morphometry.

## Introduction

Tobacco smoking in the form of cigarettes continues to be the leading cause of preventable illness and mortality in the world (1). China, the largest producer of tobacco, is estimated to contain 311 million individuals who are current smokers, with 295 million men and 16 million women, respectively (2). Chronic smoking is known to correlate with a series of diseases including stroke, lung cancer, hepatocellular carcinoma, and vascular dysfunctions (3–7). Related neuroimaging studies also suggested that the numerous toxic chemicals contained in a cigarette, especially nicotine, could promote potential brain afflictions in chronic cigarette smokers (8, 9).

Apart from these serious public health problems, 78% of smokers who expressed willingness to quit smoking reported a relapse situation in China, and the percentage in America is currently 80% (10, 11), indicating the ineffectiveness of existing cessation treatments. Poor treatment outcome for smoking cessation may result from a lack of awareness of the mechanism behind smoking addiction and the available biomarkers that characterize heavy smokers (12, 13).

Structural magnetic resonance imaging (sMRI), which visualizes the central neural system with high-resolution in a non-invasive way, provides indispensable spatial information in the procedure of identifying such biomarkers (i.e., discriminative brain regions). For the past decade, univariate approaches such as voxel-based morphometry (VBM) have continuously been applied on sMRI studies (14, 15). In these studies, morphological abnormalities are consistently discovered in heavy smokers in some brain regions, including the prefrontal cortex, anterior cingulate cortex, thalamus, and the insula (9, 14–18).

Despite the accumulative results reported, VBM, as a traditional univariate approach, has its inherent limitations in identifying spatial patterns that exist in a certain population (e.g., a group of heavy smokers). Generally, VBM has the multiple comparisons problem (19), which causes a loss of sensitivity and overlooks the dependency of the focal set of voxels in localizing informative regions relevant to specific brain abnormalities (20). Multi-voxel pattern analysis (MVPA), which utilizes a multi-variate technique and is driven by machine learning algorithms, provides a sensitive and different approach in identifying group-wise differences. MVPA takes multiple voxels into account and considers patterns across a group of voxels that may respond weakly but consistently differently between conditions (21, 22). Therefore, MVPA can be sensitive in distinguishing different experimental conditions. MVPA has become favorable in neuroimaging research for its ability to detect subtle anatomical differences (23–25).

The field of utilizing MVPA to discover structural abnormalities in heavy smokers remains less active. Notably, in an sMRI study based on support vector machine (SVM), the authors (26) used the mean gray matter volumes (GMVs) of 1,024 self-defined brain regions as input features to the SVM to identify the most discriminative regions by finding the most informative features in the SVM. Using average brain region GMVs as features may be an over simplistic way to characterize the anatomical structure of the neural system. The subtle difference within the same self-defined region may be overlooked. In this study, in order to discover smaller regions that reflect the experimental conditions of interest, we used a searchlight algorithm by moving a searchlight window through the volume of the brain, to sample the gray matter values of voxels as the input features to our linear classifier (SVM). A searchlight algorithm is able to preserve subtle differences between a group of voxels while a linear SVM can detect such subtle differences by efficiently defining a boundary that maximally separates two classes (e.g.,: heavy smokers and healthy non-smokers) of samples.

With regard to the peer studies on smoking addiction and the limitations of the current univariate approach, in this study, we aimed to: (1) explore the most discriminative gray matter regions between heavy smokers and otherwise healthy controls (HCs) with a data-driven MVPA technique and sMRI data; and (2) explore the methodological difference between MVPA and VBM. We hypothesized that MVPA would be a more sensitive method in locating group-wise brain differences than VBM.

## Methods

### Participants

The study adhered to the Declaration of Helsinki and was approved by the Medical Ethics Review Board of Zhongnan Hospital of Wuhan University. After a complete description of the study, written informed consent was obtained from the subjects.

Sixty-eight right-handed subjects (39 heavy smokers and 29 healthy non-smoker control subjects) were recruited via advertisement flyers and referrals for MRI studies. All subjects were screened for psychiatric and non-psychiatric medical disorders using the Mini International Neuropsychiatric Interview (27). All participants underwent an interview session followed by an inclusion procedure. The inclusion criteria for both groups were: the absence of any history of medical (e.g., cardiac disease) or neurological (e.g., stroke) disorders, intellectual disability, drug abuse or dependence (other than nicotine dependence for heavy smokers), or psychiatric disease; none of the subjects reported the daily consumption of alcohol or experienced social consequences secondary to alcohol intake. Heavy smokers were defined as those who met the DSM-IV criteria for nicotine dependence, had smoked at least 20 cigarettes daily in the past 5 years, and had no period of smoking abstinence longer than 3 months. The severity of the heavy smokers' nicotine addiction was measured using the Fagerström Test for Nicotine Dependence (28). The healthy control subjects in this study were defined as those who had a history of smoking no more than five cigarettes in a lifetime.

Data from two heavy smokers and one non-smoker were excluded for not meeting the above criteria. As a result, 37 heavy smokers (male: 30; female: 7) and 28 healthy non-smoking control subjects (male: 21; female: 7) were included in this study. More detailed information on the subjects in each group is presented in Table 1. Demographic data were compared between the two groups by using two-sample *t*-tests or a Chi-square test in the Statistical Package for Social Science, version 19 (SPSS Inc., USA). The threshold level of significance was set as *p* < 0.05.

**Table 1:** Demographic and clinical characteristics of the heavy smokers and HCs.

**Measure**	**HS**	**HC**	***P*-value**
Number	37	28	
Age (SD)/years	47.18 (7.22)	43 (9.62)	0.96^a^
Gender (male/female)	29/8	20/8	0.77^b^
Years of education (SD)	9.24 (2.16)	11.67 (4.72)	0.36^a^
Lifetime cigarette usage (years)	25.34 (9.23)	–	–
Age at first cigarette use (years)	21.02 (5.38)	–	–
Average cigarette per day	35.13 (10.70)	–	–
FTND score	8.89 (0.68)	–	–

*Data are presented as mean (standard deviation). HS, heavy smokers; HC, healthy controls. FTND, Fagerström Test for Nicotine Dependence*;

a *, two-sample two tailed t-test*;

b *, Chi-square test*.

### MR Acquisition

All participants underwent a high-resolution 3dn-MTC T1-weighted structural scan using a Siemens Trio 3.0-Tesla MR scanner (Erlangen, Germany) with a standard birdcage head coil. sMRI images were obtained using a MPRAGE pulse sequence with the following parameters: repetition time = 25 ms; echo time = 4.51 ms; flip angle = 25; acquisition matrix = 256 × 256; and slice thickness = 1 mm with a 1 mm gap.

### Image Preprocessing

The raw DICOM images of sMRI were converted to the NIFTI format using MRIcron (University of South Carolina, Columbia, SC, USA, http://www.mricro.com). The following preprocessing steps were then performed to obtain gray matter (GM) maps using VBM8 toolbox (http://dbm.neuro.unijena.de/vbm) in SPM8 (Version 6313, Wellcome Department of Imaging Neuroscience, London, UK, http://www.fil.ion.ucl.ac.uk/spm) on MATLAB R2013a. Firstly, the sMRI images were registered to the Montreal Neurological Institute (MNI) stereotactic space and resampled to a 1.5 mm isotropic voxel spacing. Secondly, the co-registered images were segmented into three types of tissues, namely GM, white matter, and cerebrospinal fluid. Thirdly, a study-specific template was created using the high-dimensional Diffeomorphic Anatomical Registration Through Exponentiated Lie Algebra (DARTEL) algorithm and with the predefined templates in the VBM8 toolbox. Next, in order to preserve the total volume of each brain tissue, the segmented images were modulated using non-linear deformation which can compensate for the effect of spatial normalization. This step multiplied the spatially normalized gray matter by its relative volume before and after spatial normalization (29). At last, the images were smoothed with an 8-mm full-width-half-maximum Gaussian kernel.

### MVPA

An MVPA technique combining a searchlight algorithm and a linear SVM was used to classify prominent regions that distinguished heavy smokers from HCs. Generally, by moving a searchlight region through the brain volume, one can continuously map the information content regarding the experimental conditions of interest in the brain (30). The procedure of our MVPA method is as follows. The smoothed GM maps computed from the data preprocessing step were used as inputs in the MVPA. Firstly, at each voxel (Vi) of the GM images in the normalized space, a three-dimensional cubical region size of 3 × 3 × 3 centering at Vi was identified. For each subject, the gray matter volume values of all 27 voxels (at a specific voxel position) were extracted and converted into a high-dimensional vector and used to construct the feature matrix. To train and test the SVM, a leave-one-out (LOO) cross-validation (CV) strategy was adopted, which excludes one subject as a testing set each time and trains the classifier using the remaining subjects. As a result, two feature matrices M_F1*S_ and M_F2*S_ were retrieved as the training set and testing set (F1 = 64, F2 = 1, S = 27), respectively. F1 and F2 indicate the number of subjects in the two sets respectively, and S indicates the number of voxels of the feature matrix. Next, the training set was fed into the SVM implemented in the LIBSVM toolbox (http://www.csie.ntu.edu.tw/~cjlin/Libsvm). In each training set, a nested 5-fold CV was applied to determine the optimized parameter C (regularization) and g (gamma for radius basis function) for testing. That is, each time one-fifth of the training set was selected as a testing sample and the classifier was built upon the remaining data. Parameters that produced the highest accuracy across these 5-folds was identified as the optimized C and g. The identified C and g were then used in the corresponding testing procedure. Finally, the accuracy of the trained classifier was assigned to the chosen voxel Vi. After repeating this procedure on every voxel, a three-dimensional accuracy map denoting the classification ability between heavy smokers and HCs for all voxels was obtained.

To evaluate the statistical significance of the experimental results, we converted the accuracy map to a *p*-value map under an assumption of binomial distribution. Detailed information about the conversion procedure can be found in another publication (25). A connected domain algorithm was conducted on the *p*-value map to produce clusters with significant predictive power, whose threshold was set at *p* < 0.001 with more than 50 adjacent voxels (24).

### *Post hoc* Analysis of MVPA Analysis Results

To further explore the difference of GMVs between heavy smokers and HCs, a *post hoc* analysis was carried out, with which a further VBM analysis was conducted within the brain clusters detected by MVPA. The analysis was corrected for multiple comparisons using family wise error (FWE) at the cluster level (*p* < 0.05). To do this, firstly the GMVs of the brain regions detected by the MVPA were extracted using the MarsBar toolbox (http://www.mrc-cbu.cam.ac.uk/Imaging/marsbar.html). Then, with age and gender as co-variates, voxel-wise two independent samples *t*-tests were further performed in these regions to determine the significant differences between heavy smokers and HCs (*p* < 0.05, FWE-corrected, two-tailed).

### Whole Brain VBM Analysis

In order to compare the methodological differences between MVPA and VBM, a whole brain VBM analysis was conducted. With age and gender as co-variates, the whole brain GM maps of heavy smokers and HCs were fed to VBM. The same threshold values of *post hoc* analysis were applied in order to conduct a comparison between whole brain VBM analysis and MVPA. Discriminative regions discovered by the whole brain VBM were defined as clusters with a cluster size > 50 voxels and a *p*-value < 0.001.

### ROC Analysis

To evaluate the ability of MVPA or VBM in distinguishing the smokers from the healthy controls, we performed ROC analysis based on the brain regions with significant differences. Firstly, we used the average gray matter values of brain regions as features to establish an SVM model using 5-fold cross validation. Then the ROC analysis was performed with the predicted probability of each participant in the procedure of cross validation. The area under the ROC curve (AUC) denotes the ability of classifying heavy smokers from healthy controls.

## Results

### Demographic Characteristics

Heavy smokers did not differ significantly with HCs in terms of age (*p* = 0.96), years of education (*p* = 0.36), or gender ratio (*p* = 0.77). For heavy smokers, the average FTND score was 8.89 ± 0.68 (range, 8–10; median, 9) and the average number cigarettes per day was 35.13 ± 10.70, which indicated a relatively high dependence on cigarettes. Detailed demographic information for both groups can be found in Table 1.

### MVPA and *post hoc* Analysis

**Figure 1:**
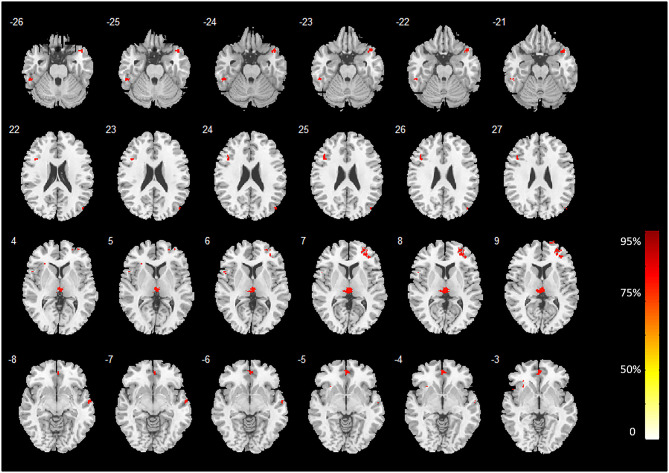
Brain regions with high classification accuracy identified by MVPA. The color bar indicates the classification accuracy values of the whole brain GM voxels. The image is displayed in the neurologic convention, with the left side corresponding to the left brain.

The discriminative regions recognized by our MVPA technique without covariates regression before classifier training are shown in Figure 1. Our proposed technique yielded a voxel-wise accuracy of at least 81% for classifying heavy smokers from HCs. Several cortical and subcortical regions demonstrated a strong classification ability with GM differences between heavy smokers and HCs. These regions were primarily located at the temporal cortex and prefrontal cortex, occipital cortex, thalamus (bilateral), insula (left), anterior and median cingulate gyri (ACG and MCG), and precuneus (left).

**Figure 2:**
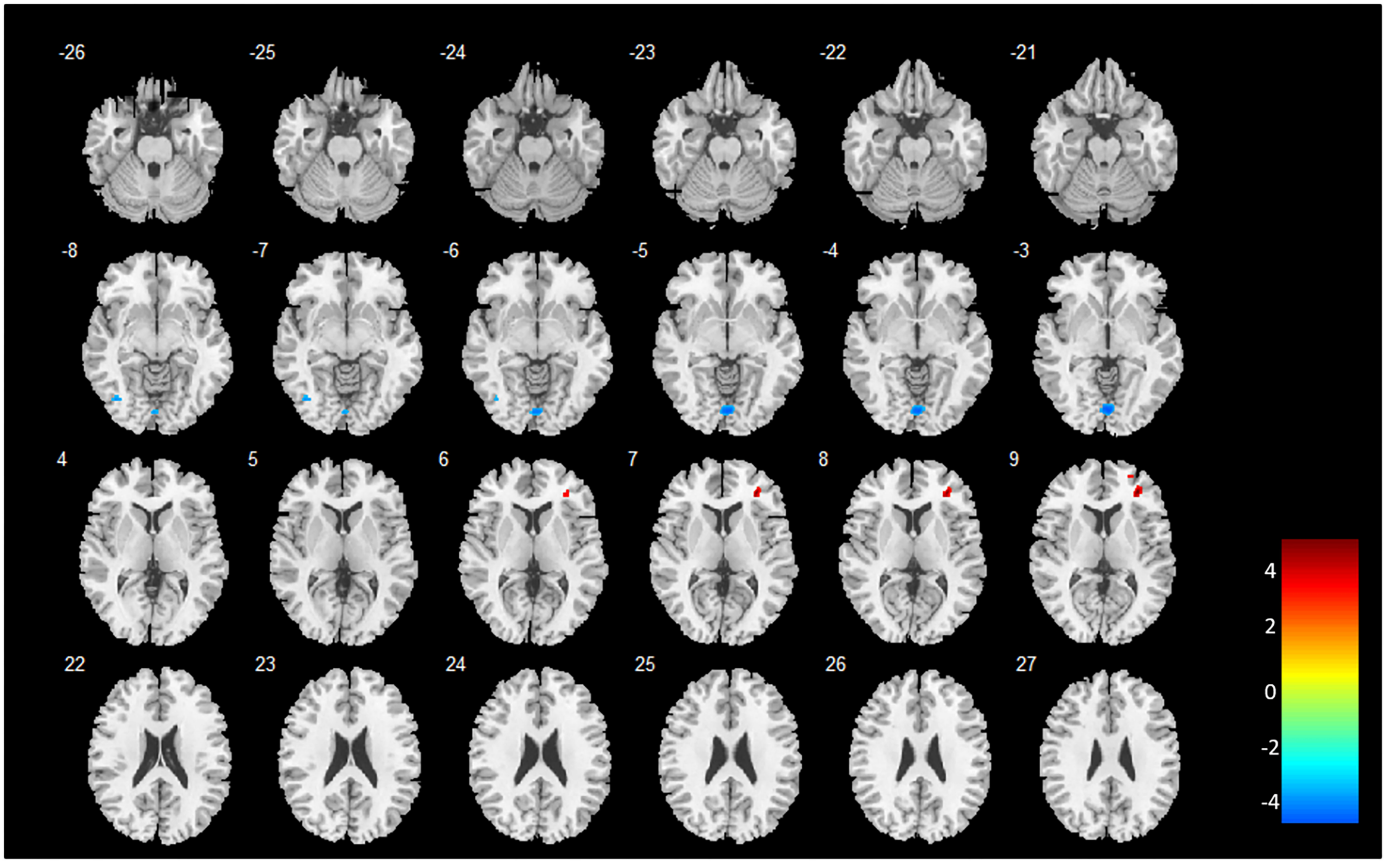
Brain regions identified by whole brain VBM analysis. The color bar indicates the peak t-values of the maximum voxel found in the extracted cluster. A positive *t*-value indicates that the GM volume in HS is significantly smaller than HC in a specific region while a negative value indicates the opposite. The image is displayed in the neurologic convention, with the left side corresponding to the left brain.

Two-sample *t*-tests (*post hoc* analysis) of GMVs revealed that 16 out of the 18 brain regions identified by MVPA demonstrated a significant GMV decrease in heavy smokers as compared to healthy controls (*p* < 0.05, FWE-corrected, two-tailed). Notably, the inferior temporal gyrus (left) and cerebellum were found with a significantly increased GMV in heavy smokers than in HCs. The peak accuracy values of these clusters and the corresponding *t*-values which were derived from the *post hoc* analysis are reported in Table 2. The peak *t*-value was defined as the maximum *t*-value found in the extracted cluster. A positive *t*-value indicated that the GM volume in HS was significantly smaller than HC in a specific region while a negative value indicated the opposite. The brain regions recognized by VBM are shown in Figure 2.

**Table 2:** Brain regions with high predictive accuracy identified by MVPA and the corresponding *post-hoc* (VBM) results, which was corrected for multiple comparisons using family wise error (FWE) at the cluster level (*p* < 0.05).

**Brain regions (AAL)**	**Cluster size (voxels)**	**Peak MNI coordinates**			**Peak *t*-value**
		**X**	**Y**	**Z**	
Temporal_Pole_Mid_R	78	42	7.5	−37.5	3.69
Occipital_Inf_L	64	−36	−73.5	−0.5	−3.87
Calcarine_L	525	1.5	−79.5	−3	−4.62
Frontal_Med_Orb_L	89	−13.5	63	−3	3.91
Postcentral_R	622	24	−37.5	81	4.17
Temporal_Inf_L	143	−54	−39	−19.5	−4.12
Lingual_L	197	1.5	−78	−1.5	−4.32
Cuneus_L	317	−1.5	−97.5	18	4.19
Angular_R	449	45	−75	40.5	3.89
Occipital_Sup_L	294	−13.5	−99	31.5	4.30
Frontal_Mid_R	217	31.5	42	9	4.50
Parietal_Sup_R	98	15	−72	70.5	3.95

*AAL, anatomical automatic labeling; MNI, Montreal Neurological Institute; L, left hemisphere; R, right hemisphere; acc, accuracy; HS, heavy smokers; HC, healthy controls. Peak t-value, post-hoc (VBM) results, conducted on brain regions detected by MVPA and corrected for multiple comparisons using family wise error (FWE) at the cluster level (p < 0.05). Positive t-value indicates GM volume in HS is significantly smaller than HC while negative value indicates the opposite*.

**Figure 3:**
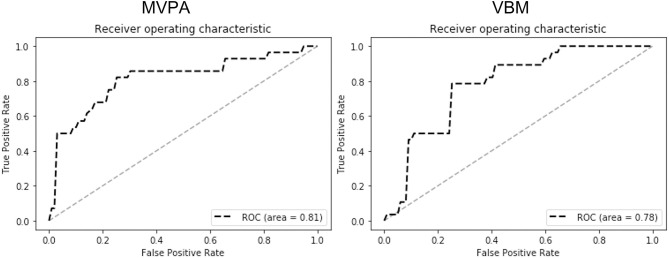
The ROC analysis results of MVPA (left) and VBM (right). The results (AUC, 0.81 vs. 0.78; sensitivity, 0.82 vs. 0.79; specificity, 0.77 vs. 0.77; PPV, 0.72 vs. 0.71; NPV, 0.85 vs. 0.82) indicate that MVPA outperformed VBM in differentiating the smokers from healthy controls by using the GMVs of the detected brain regions as the features.

The ROC analysis results are shown in Figure 3. The ROC results indicate that MVPA outperformed VBM in differentiating the heavy smokers from the HCs (AUC, 0.81 vs. 0.78; sensitivity, 0.82 vs. 0.79; specificity, 0.77 vs. 0.77; positive predictive value (PPV), 0.72 vs. 0.71; negative predictive value (NPV), 0.85 vs. 0.82).

### VBM Analysis

As summarized in Table 3, whole brain VBM analysis revealed that six brain regions demonstrated a significant decrease or increase of GMV in heavy smokers than in HC. These regions were located in the occipital, precuneus lingual, and cerebrum. The peak *t*-value was defined as the maximum *t*-value found in the extracted cluster. A positive *t*-value indicated that the GM volume in HS was significantly smaller than HC in a specific region while a negative value indicated the opposite.

**Table 3:** Brain regions detected by VBM with a significant decrease or increase GMV in heavy smokers than HC.

**Brain regions (AAL)**	**Cluster size (voxels)**	**Peak MNI coordinates**			**Peak *t*-value**
		**X**	**Y**	**Z**	
**HS** **<** **HC**					
Right cerebrum	129	31.5	40.5	9	5.313
Left cerebrum	73	−13.5	−99	31.5	3.63
Occipital_Sup_R	100	21	−93	46.5	3.88
Precuneus_L	50	−12	−49.5	51	3.93
**HS>HC**					
Occipital_Inf_L	58	−40.5	−70.5	−9	−3.64
Lingual_L	355	−1.5	−79.5	−1.5	−4.39

*AAL, anatomical automatic labeling; MNI, montreal neurological institute; L, left hemisphere; R, right hemisphere; HS, heavy smokers; HC, healthy controls. t-value, positive t-value indicates GM volume in HS is significantly smaller than HC while negative value indicates the opposite. The cluster size is more than 50 voxels and the threshold is set as p < 0.001, uncorrected*.

## Discussion

Traditional univariate studies prior to this research have constantly discovered that heavy smokers shared similar GM alterations in the cingulum, thalamus, cerebellum, prefrontal gyrus, and precuneus (9, 15–18, 31). In this study, these regions were identified by MVPA as discriminative regions between heavy smokers and HCs. In a following *post hoc* analysis on these particular regions, our analysis results also demonstrated significant GM decrease in several areas including the temporal cortex, prefrontal cortex, thalamus, ACG and MCG, and precuneus in heavy smokers, indicating a strong correlation between GM alteration in regional GM deficiency and chronic smoking. In addition, our MVPA result also revealed that regions (insula, cerebellum) which were seldomly reported in traditional VBM analysis, may participate in the chronic smoking mechanism. The resulting contrast between MVPA and VBM may indicate the methodological differences between these two methods.

In our study, the bilateral thalamus were identified by MVPA with high accuracy. Particularly, in the *post hoc* analysis, the right thalamus was found to have significant GMV loss in heavy smokers. The observation of GMV deficiency in heavy smokers may primely result in the cognitive impairment reported in several studies (8, 17), as the thalamus relays information between the cerebral and different subcortical cortexes (32). In addition to cognitive impairment, the thalamus has been identified as a brain region with a relatively high density of nicotine acetycholine receptors (α4β2^*^ nAChRs) (23, 33, 34). Frequent nicotine binding activities could be the leading reason for the GMV decrease in the thalamus. Such a finding also provides insight for formulating more effective withdrawal treatment. In a related Positron Emission Computed Tomography (PET) study (33), the author suggested that maintaining α4β2^*^ nAChRs in the desensitization state may be a possible way to alleviate withdrawal symptoms.

The cingulum, bidirectionally connected with the medial temporal lobes, was another region widely reported to be associated with smoking addiction in univariate studies. In this study, the bilateral cingulum could discriminate heavy smokers from HCs with at least an accuracy of 75%. In the corresponding *post hoc* analysis, both the ACG and MCG demonstrated significant GM loss in heavy smokers. As a region connected to sites repeatedly implicated in cognitive control, the ACG is involved in executive function behaviors, such as inhibitory control and conflict resolution (35), which may play an important part in the process of quitting smoking.

The prefrontal area was another discriminative region identified by MVPA with high accuracy. Especially, in the *post hoc* analysis, the inferior and middle areas were found with significant GMV loss in heavy smokers as compared to HCs. As the prefrontal cortex is associated with concentration, emotion, and other higher brain functions, our findings with the prefrontal cortex may partially explain the decreased attention and impairment of working memory reported in heavy smokers (36–39).

Among other regions detected by MVPA, the insula presented relatively high predictive accuracy and stood out as a discriminative region. Acting as a critical neural substrate for addiction including nicotine dependence (40), the insula plays a potential role in the decision-making task that is associated with relapse to drug use (41) and cue-induced drug urges (14, 42, 43). However, the insula was not reported in our whole brain VBM analysis nor in other univariate sMRI studies. In contrast, our finding of the insula was in accordance with another multi-variate sMRI study deploying SVM (26). The fact that the insula along with the ACG are part of the salience network (SN) (44), a network that mediates one's subjective feelings, is thought to be one of the solutions to developing effective treatment plans. A previous fMRI study discovered that damage to the insula could disrupt addiction to cigarette smoking which in turn may help improve smoking cessation outcomes (40). Besides, evidence of the insula's role in mediating addiction urges may partially justify why the insula stands out as a discriminative region.

Notably, nicotine binding activity in the cerebellum, a region that was identified by our MVPA method with high accuracy (82%) has also been reported in several biochemical studies (45, 46). Heavy smokers showed a greater density of nicotine binding in the cerebellum as compared to non-smoking individuals (47), and brain blood flow in the cerebellum was increased by cigarette smoking (45, 46). This evidence lends support to the fact that decreased cerebellar GMV is associated with smoking addiction. However, like the insula and other regions, significantly lower GMV was not found in the cerebellum in heavy smokers more than in healthy controls in the whole brain VBM analysis. Moreover, as indicated by sMRI studies deploying ML techniques (26, 48), the insula and cerebellum were found to participate in the mechanism of chronic smoking. Such differences may be attributed to the fundamental difference between MVPA and traditional univariate analysis.

To be specific, MVPA fixes what was considered a disadvantage in traditional univariate methods. Conventional univariate analysis methods try to separately find voxels that show a statistically significant response to the experimental/physical conditions by deriving the average of the chosen voxels in all subjects, which assumes that the covariance across neighboring voxels is not informative about the experimental conditions under examination (49). Such assumption leads to the following inadequacies. Firstly, low response voxels that still carry important information could therefore be dismissed. Secondly, a consistent spatial pattern is neglected by simply averaging all voxels. MVPA uses a different solution to boost accuracy. Instead of uniformly averaging all voxels in VBM, MVPA assigns a weighted average in different conditions. This operation allows MVPA to discover spatial patterns neglected by VBM. Further, MVPA tries to optimize these weights by involving data-driven machine learning techniques. The evidence that the regions showing correlation to chronic smoking also implied that MVPA could be more sensitive than conventional univariate methods. In this view, MVPA can be better than VBM in discovering specific patterns in the chronic smoking population.

The study still presents the following limitations. Firstly, the sample size in the current study is relatively small. In a future study, more data should be collected in order to construct a more robust classifier and to verify our findings. Secondly, a more comprehensive dataset combining different modalities, such as fMRI and diffusion tensor imaging, would be helpful in exploring the mechanism of discriminative ability of such regions in heavy smokers.

In this study, MVPA was applied to structural MRI to identify the brain regions in discriminating heavy smokers from healthy individuals. The anatomical deficiency in heavy smokers was mainly discovered in the insula, ACG, MCG, prefrontal cortex, precuneus, and cerebellum, which are highly involved in the addiction of chronic smoking. These findings in accordance with previous results (45, 47, 48, 50) might provide insights for understanding the mechanism of chronic smoking and effective cessation treatment. Such insights also indicate the potential of using MVPA in future neuroimaging research. Moreover, the comparison between VBM and MVPA revealed that MVPA can be efficient in locating brain discriminative regions which were neglected by VBM.

## Data Availability Statement

The data analyzed in this study is subject to the following licenses/restrictions: The data for this study are not publicly available because Zhongnan Hospital of Wuhan University, the center from which the data were collected, does not agree to make the data publicly accessible. Requests to access these datasets should be directed to huangb@szu.edu.cn.

## Ethics Statement

The studies involving human participants were reviewed and approved by Medical Ethics Review Board of Zhongnan Hospital of Wuhan University. Written informed consent for participation was not required for this study in accordance with the national legislation and the institutional requirements.

## Author Contributions

YY, JZ, GD, and GW: conception and design. GD and GW: administrative support. GW: provision of study materials or patients. YY, JZ, GD, GW, and SW: collection and assembly of data. YY, JZ, GW, and SW: data analysis and interpretation. All authors: manuscript writing. All authors final approval of manuscript.

## Conflict of Interest

The authors declare that the research was conducted in the absence of any commercial or financial relationships that could be construed as a potential conflict of interest.
